# Temporal trend of food consumption indicators of adolescents monitored by Primary Health Care services of the Brazilian National Health System, 2015-2019

**DOI:** 10.1590/S2237-96222025v34e20240893.en

**Published:** 2025-08-04

**Authors:** Joana Brant de Carvalho, Andressa Freire Salviano, Bárbara Hatzlhoffer Lourenço, Luciana Yuki Tomita

**Affiliations:** 1 Universidade Federal de São Paulo, Escola Paulista de Medicina, Departamento de Medicina Preventiva, São Paulo, SP, Brazil; 2 Universidade de São Paulo, Faculdade de Saúde Pública, Programa de Pós-Graduação Nutrição em Saúde Pública, São Paulo, SP, Brazil; 3 Universidade de São Paulo, Faculdade de Saúde Pública, Departamento de Nutrição, São Paulo, SP, Brazil

**Keywords:** Eating, Adolescents, Food and Nutritional Surveillance, Health Information Systems, Time Series Studies, Ingestión de Alimentos, Adolescentes, Vigilancia Alimentaria y Nutricional, Sistemas de Información en Salud, Estudios de Series Temporales.

## Abstract

**Objective:**

To evaluate the temporal trend of dietary practices of adolescents monitored in Primary Health Care from 2015 to 2019.

**Methods:**

This is a time series study with microdata on adolescent food consumption markers (n=674,280 records, mean age: 14.9 [standard deviation: 3.0] years, 65.0% girls) held on the Food and Nutrition Surveillance System. Annual prevalence of each marker was calculated, in addition to the following indicators: combined consumption of beans, fruit, vegetables and greens; and ultra-processed food. Prais-Winsten regression was used to calculate the annual increase rate, according to sex, age group (10-14 years, 15-19 years) and macro-regions. Increasing or decreasing trends were denoted by a positive or negative annual increase rate with p-value<0.05.

**Results:**

The prevalence rates of consumption of beans, fruit, vegetables and legumes in 2019 were 83.0%, 69.0% and 65.0%. The combined indicator of beans, fruit, vegetables and legumes was found in 48.0% of adolescents in 2019, with a rising trend in Brazil as a whole (annual increase rate: +4.0%, 95% confidence interval [95%CI 0.4; 6.6]), in the 15-19 age group and in the Southeast region. In 2019, markers of ultra-processed food consumption were frequent among adolescents (burgers and sausages: 45.0%, sugar-sweetened drinks: 65.0%, instant noodles/snacks: 46.0%, sweets: 55.0%). The prevalence of the ultra-processed food indicator in 2019 was 23.0%, with a rising trend nationally (annual increase rate: +7.0%, 95%CI 2.9; 11.7) and in the South and Southeast macro-regions.

**Conclusion:**

Although some healthy indicators have increased, prevalence of ultra-processed food consumption among adolescents is high.

## Resumen

**Objetivo:**

Evaluar la tendencia temporal de las prácticas alimentarias de los adolescentes monitoreados en Atención Primaria de Salud en el período 2015-2019.

**Métodos:**

Se trata de un estudio de series temporales con microdatos sobre marcadores de consumo de alimentos en adolescentes (n=674.280 registros, edad media: 14,9 [desviación estándar: 3,0] años, 65,0% mujeres) en el Sistema de Vigilancia Alimentaria y Nutricional. Se calcularon las prevalencias anuales de cada marcador, además de los indicadores: consumo combinado de frijoles, frutas, verduras y hortalizas; y alimentos ultraprocesados. Se utilizó la regresión de Prais-Winsten para calcular la tasa de incremento anual, según sexo, grupo de edad (10-14 años, 15-19 años) y macrorregiones. Las tendencias crecientes o decrecientes se denotaron por una tasa de incremento anual positiva o negativa con un p-valor<0,05.

**Resultados:**

La prevalencia de consumo de frijoles, frutas, verduras y legumbres en el año 2019 fue de 83,0%, 69,0% y 65,0%. El indicador combinado de frijoles, frutas, verduras y hortalizas se presentó en el 48,0% de los adolescentes en 2019, con tendencia creciente en el país (tasa de incremento anual: +4,0%, intervalo de confianza del 95% [IC95% 0,4 6,6]), entre los 15-19 años y en la región Sureste. En 2019, el consumo de marcadores alimentarios ultraprocesados ​​fue frecuente entre los adolescentes (hamburguesas y salchichas: 45,0%, bebidas azucaradas: 65,0%, fideos instantáneos/snacks: 46,0%, dulces: 55,0%). La prevalencia del indicador de alimentos ultraprocesados ​​en 2019 fue de 23,0%, con tendencia creciente a nivel nacional (tasa de incremento anual: +7,0%, IC95% 2,9; 11,7) y en las macrorregiones Sur y Sudeste.

**Conclusión:**

Aunque algunos indicadores saludables han aumentado, la prevalencia del consumo de alimentos ultraprocesados ​​entre los adolescentes es alta.

**Ethical aspects**

This research respected ethical principles, having obtained the following approval data::

Research Ethics Committee: Universidade de São Paulo

Opinion number: 4,172,787

Approval date: 24/7/2020

Certificate of Submission for Ethical Appraisal: 35480520.2.0000.5421

Informed Consent Form: Exempted.

## Introduction

Consumption of insufficient amounts of fruit, vegetables and beans and excessive amounts of ultra-processed foods, such as soft drinks, sweets and treats, snacks and fast food, has been observed in recent decades among adolescents. This pattern points to the risk of replacing traditional diets with obesogenic diets, low in fiber and high in calories, sodium, fat and added sugar (1-3). The association between intake of ultra-processed foods and increased risk of adverse health events has been demonstrated. Consumption of these foods has been related to more than 30 health problems, especially cardiometabolic ones, including obesity, diabetes, hypertension, dyslipidemia, as well as poor sleep, cancer, other chronic diseases and mortality due to all causes (4). In children and adolescents, a correlation has been observed with early menarche, mental health problems and dental caries (5-7). This mainly affects low- and middle-income countries and is a global public health challenge (8-9).

In Brazil, the population’s food consumption is continuously monitored by Primary Health Care (PHC) using food consumption markers held on the Food and Nutrition Surveillance System (SISVAN) (10). These markers are instruments for screening dietary practices and food group intake and assess food consumption on the previous day in order to avoid memory bias. It is an appropriate tool at the individual level to guide production of health care by different health professionals. At the population level, availability of this information allows monitoring of key qualitative aspects of the diet over time, which can inform the proposal of policies for health promotion (10).

Given the risks associated with poor nutrition, this study aimed to evaluate the temporal trend of food consumption indicators of Brazilian adolescents attending PHC and recorded on the SISVAN. There was interest in exploring combinations of food consumption markers and investigating variations according to sex, age groups and Brazilian regions.

## Methods

### Study design and data sources

This time series study used microdata derived from aggregation of individual information on food consumption markers held on the SISVAN. A total of 674,280 records of adolescents (10-19 years of age) between 2015 and 2019 were used. The period used included the year in which the food consumption marker form was included on the SISVAN in the light of the recommendations of the Dietary Guidelines for the Brazilian Population, up to the pre-pandemic period and the time when the databases were requested at the beginning of the research project.

The food consumption markers were proposed based on the recommendations of the Dietary Guidelines for the Brazilian Population and included nine questions on dietary practices and intake of seven food groups. For this study, only data on food consumption were used, with possible answers being “yes”, “no” or “don’t know” for consumption on the previous day of the following foods: beans; fresh fruit excluding fruit juices; vegetables and legumes, except tubers; hamburgers and sausages; sugar-sweetened drinks; instant noodles, packaged snacks or salted biscuits; filled cookies, sweets or treats (10). The internal structure of the SISVAN food consumption marker form showed evidence of validity for assessing two dietary factors (healthy and unhealthy), with stability of measurement characteristics between Brazilian macro-regions and age groups over time (11).

### Data management and indicator building

**Figure 1:**
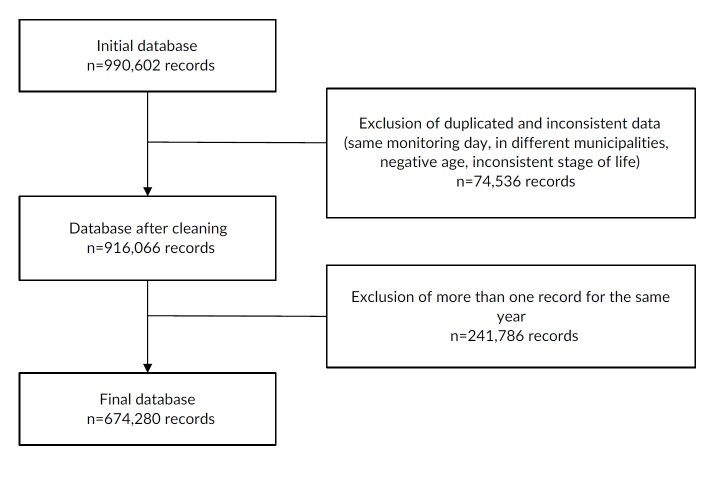
Cleaning of data on markers of adolescent food consumption in relation to Primary Health Care. Brazil, 2015-2019

The databases underwent a cleaning stage, in which duplicate data for the same individual in the same municipality and on the same day, data for the same individual in different municipalities, and inconsistent data such as negative age or life stage not corresponding to the completed form were identified and excluded. After data cleaning, only the first record of the year for each adolescent was considered. In the period 2015-2019, out of 990,602 records in the age group of interest, 74,536 duplicate or inconsistent records were excluded. By maintaining only one record per adolescent per year, 241,786 observations were eliminated, which resulted in 674,280 records for the time series analyses (Figure 1).

Prevalence of adolescents with positive responses to the markers on previous day consumption was calculated from 2015 to 2019. In addition to the markers, food consumption indicators were created through groupings: (i) combined consumption of beans, fruit, vegetables and greens; (ii) consumption of ultra-processed food (sausages, sugar-sweetened drinks, instant noodles, salted biscuits, sweets, treats and filled cookies); and (iii) healthy consumption, whereby individuals who consumed all the healthy eating markers and no unhealthy markers on the previous day were considered, in light of the recommendations of the Dietary Guidelines for the Brazilian Population: “make unprocessed or minimally processed foods the basis of your diet” and “avoid ultra-processed foods”.

### Data analysis

Data analysis was performed using Prais-Winsten regression, with calculation of the rate of annual increase in the prevalence of markers and indicators of food consumption among adolescents. This approach considered serial correlation of measurements, that is, the dependence of a serial measurement on its own values ​​at previous times, commonly observed in population data. From the coefficients estimated in the Prais-Winsten regression, the annual increase rates and confidence intervals (95%CI) were obtained using the following formula (12):


$$\text{Annual increase rate}=[–1+10^{\beta }]*100$$

95%
CI lower=[–1+10β
mín]∗
100

95%
CI upper=[–1+10β
máx]∗
100

β=Prais-Winsten regression coefficient

The data were analyzed at the national level, according to sex (male, female), age group (10-14 years old; 15-19 years old) and also by Brazil’s macro-regions (North, Northeast, Midwest, South and Southeast). Stata version 17.0 was used for this purpose. Interpretation of the temporal trend was given by the annual increase rate values ​​and the level of statistical significance: increasing when the annual increase rate values ​​were positive, and decreasing when negative, along with a 5.0% statistical significance level. Arrows were used to represent the temporal trend results: “↗” when rising, “↘”when falling and “↔” when stationary. 

Use and management of the databases used in this study are in line with the normative criteria of National Health Council Resolution No. 738, dated November 7, 2024 (13).

## Results

A total of 674,280 records of adolescents monitored in PHC between 2015 and 2019 were obtained, with a mean age of 14.9 (standard deviation: 3.0) years, and 65.0% were female. Prevalence of bean consumption on the previous day was 83.0% in 2019 for Brazil as a whole, with no significant temporal variation nationally or according to sex, age group or region (Table 1). Fruit consumption remained stable in Brazil and for both sexes and age groups, with national prevalence of 69.0% in 2019. The only significant reduction occurred in the Northern region (2015: 70.0%, 2019: 65.0%; annual increase rate -1.43%, 95%CI -2.28; -0.57), while an increase was recorded in the Southeast region (2015: 65.0%, 2019: 68.0%; annual increase rate +2.30%, 95%CI 0.79; 3.84). Consumption of vegetables and legumes showed stationary trends in all stratifications investigated, with overall prevalence of 65.0% in 2019 for Brazil as a whole.

**Table 1:** Prevalence of consumption of healthy foods on the previous day and annual increase rate with 95% confidence intervals (95%CI) among adolescents, according to sex, age group and macro-region, in relation to Primary Health Care. Brazil, 2015-2019 (n=674,280)

	2015	2016	2017	2018	2019	Annual increase rate (95%CI)^a^	p-value	Trend^b^
	%	%	%	%	%			
**Consumption of beans**								
Brazil	83.0	81.0	84.0	83.0	83.0	0.26 (-0.60; 1.14)	0.407	↔
Sex								
Male	85.0	82.0	87.0	85.0	85.0	0.37 (-0.67; 1.43)	0.340	↔
Female	83.0	80.0	83.0	81.0	81.0	0.16 (-0.67; 1.00)	0.578	↔
**Age group (years)**								
10-14	83.0	80.0	84.0	82.0	82.0	0.25 (-0.74; 1.25)	0.480	↔
15-19	84.0	81.0	84.0	83.0	83.0	0.28 (-0.54; 1.10)	0.359	↔
Macro-region								
North	77.0	69.0	75.0	73.0	72.0	-0.07 (-2.44; 2.37)	0.936	↔
Northeast	86.0	83.0	84.0	84.0	85.0	-0.12 (-1.18; 0.95)	0.738	↔
Southeast	87.0	86.0	89.0	87.0	86.0	0.22 (-0.96; 1.41)	0.604	↔
South	78.0	78.0	79.0	75.0	74.0	-1.46 (-3.52; 0.65)	0.114	↔
Midwest	88.0	83.0	83.0	86.0	89.0	0.64 (-2.53; 3.91)	0.571	↔
**Consumption of fruit**								
Brazil	68.0	65.0	68.0	69.0	69.0	1.20 (-0.26; 2.68)	0.079	↔
Sex								
Male	67.0	64.0	68.0	68.0	68.0	1.32 (-0.10; 2.76)	0.060	↔
Female	68.0	65.0	69.0	69.0	69.0	1.15 (-0.36; 2.67)	0.094	↔
**Age group** (years)								
10-14	68.0	65.0	68.0	68.0	68.0	1.07 (-0.31; 2.46)	0.090	↔
15-19	68.0	65.0	69.0	69.0	69.0	1.35 (-0.20; 2.93)	0.070	↔
Macro-region								
North	70.0	65.0	67.0	65.0	65.0	-1.43 (-2.28; -0.57)	0.013	↘
Northeast	67.0	63.0	66.0	68.0	69.0	1.66 (-1.18; 4.57)	0.161	↔
Southeast	65.0	64.0	67.0	69.0	68.0	2.30 (0.79; 3.84)	0.017	↗
Sout	66.0	70.0	76.0	71.0	70.0	1.43 (-3.58; 6.71)	0.438	↔
Midwest	73.0	65.0	68.0	69.0	71.0	0.45 (-3.40; 4.46)	0.738	↔
**Consumption of vegetables and legumes**								
Brazil	63.0	62.0	67.0	66.0	65.0	1.23 (-0.99; 3.50)	0.177	↔
Sex								
Male	62.0	61.0	67.0	66.0	64.0	1.71 (-1.22; 4.72)	0.162	↔
Female	64.0	63.0	67.0	66.0	65.0	0.99 (-0.87; 2.88)	0.190	↔
**Age group (years)**								
10-14	61.0	60.0	65.0	64.0	62.0	1.28 (-1.33; 3.96)	0.219	↔
15-19	65.0	64.0	68.0	68.0	67.0	1.42 (-0.28; 3.14)	0.077	↔
Macro-region								
North	62.0	56.0	58.0	58.0	58.0	-0.71 (-3.21; 1.86)	0.441	↔
Northeast	58.0	55.0	57.0	58.0	60.0	1.55 (-0.46; 3.59)	0.092	↔
Southeast	68.0	68.0	71.0	71.0	70.0	1.21 (-0.11; 2.55)	0.062	↔
South	68.0	70.0	74.0	69.0	68.0	-0.23 (-4.18; 3.87)	0.865	↔
Midwest	71.0	68.0	69.0	73.0	72.0	1.10 (-1.25; 3.50)	0.236	↔
**Combined consumption of beans, fruit, vegetables and legumes** ^c^								
Brazil	45.0	42.0	48.0	48.0	48.0	3.48 (0.41; 6.64)	0.036	↗
Sex								
Male	45.0	42.0	49.0	49.0	48.0	4.10 (0.52; 7.80)	0.035	↗
Female	45.0	41.0	47.0	48.0	47.0	3.11 (0.21; 6.11)	0.042	↗
**Age group** (years)								
10-14	44.0	40.0	47.0	47.0	46.0	3.23 (-0.10; 6.67)	0.054	↔
15-19	46.0	42.0	48.0	50.0	50.0	3.86 (0.78; 7.03)	0.028	↗
Macro-region								
North	42.0	33.0	38.0	36.0	37.0	-0.89 (-5.51; 3.97)	0.595	↔
Northeast	44.0	38.0	41.0	42.0	45.0	1.97 (-3.92; 8.21)	0.374	↔
Southeast	46.0	47.0	52.0	55.0	53.0	4.98 (2.73; 7.27)	0.006	↗
South	43.0	46.0	53.0	49.0	49.0	3.26 (-3.32; 10.28)	0.219	↔
Midwest	53.0	46.0	47.0	52.0	55.0	2.31 (-5.20; 10.41)	0.411	↔
**Healthy consumption** ^d^								
Brazil	5.0	6.0	7.0	7.0	7.0	8.34 (3.19; 13.74)	0.014	↗
Sex								
Male	4.0	5.0	6.0	6.0	6.0	9.28 (0.60; 18.70)	0.042	↗
Female	5.0	6.0	7.0	7.0	7.0	8.13 (4.66; 11.71)	0.005	↗
**Age group (years)**								
10-14	4.0	5.0	6.0	6.0	6.0	9.27 (0.89; 18.35)	0.039	↗
15-19	6.0	6.0	7.0	7.0	8.0	8.29 (6.73; 9.88)	<0.001	↗
Macro-region								
North	5.0	5.0	7.0	6.0	7.0	9.45 (0.15; 19.62)	0.048	↗
Northeast	6.0	6.0	7.0	7.0	7.0	6.67 (4.10; 9.30)	0.004	↗
Southeast	4.0	5.0	6.0	7.0	7.0	13.61 (10.52 ;16.79)	0.001	↗
South	4.0	6.0	6.0	5.0	5.0	1.46 (-13.30; 18.74)	0.788	↔
Midwest	5.0	6.0	8.0	9.0	7.0	9.23 (-7.31; 28.70)	0.186	↔
Total	50.570	90.374	109.291	188.197	235.848			

^a^Annual increase rate calculated using the following formula [-1+(10^β^)]×100, where β is the Prais-Winsten regression coefficient; 95%CI calculated using the formulae [–1+10^βmin^]x100 e [–1+10^βmax^]x100; ^b^Trend: (↔) stationary trend, (↗) rising trend, when annual increase rate positive and p-value<0,05; and (↘) falling trend, when annual increase rate negative and p-value<0,05; ^c^Combined consumption of beans, fruit, vegetables and legumes: taking those individuals who answered “yes” to the three healthy eating markers (beans, fruit, vegetables and legumes); ^d^Healthy consumption: taking those individuals who answered “yes” to all the healthy eating markers and “no” to all the unhealthy eating markers.

Considering the indicators produced, combined consumption of beans, fruit, vegetables and legumes on the previous day showed low prevalence, ranging from 45.0% to 48.0% in Brazil as a whole between 2015 and 2019, although with a rising trend (annual increase rate +3.48%, 95%CI 0.41; 6.64). The rising trend was observed among adolescents of both sexes and aged 15 years and older. Between the country’s regions, only the Southeast showed a rising trend of 4.98% (95%CI 2.73; 7.27) and reached 53.0% in 2019. When aggregating absence of ultra-processed food, the healthy consumption indicator showed low but increasing prevalence in the country, and varied from 5.0% in 2015 to 7.0% in 2019 (annual increase rate +8.34%, 95%CI 3.19; 13.74). The positive temporal trend was similar between sexes and age groups, as well as for the North, Northeast and Southeast regions (Table 1).

For the ultra-processed food groups (Table 2), the marker of consumption of hamburgers and sausages showed an increasing temporal trend among adolescents in Brazil as a whole, reaching 45.0% in 2019 (annual increase rate +4.20%, 95%CI 2.18; 6.26). Similarly, there was a rising trend in both sexes, age groups and in the Southeast region. Consumption of sugar-sweetened drinks remained stable at the national level, with a prevalence rate of 65.0% in 2019. Female adolescents (annual increase rate -0.85%, 95%CI -1.36; -0.33) and residents of the Northern region (annual increase rate -2.66%, 95%CI -3.96; -1.35), Southeast region (annual increase rate -1.46%, 95%CI -1.64; -1.28) and Southern region (annual increase rate -2.12%, 95%CI -2.44; -1.79) showed a reduction in the prevalence of this marker. Despite the stable trend in Brazil as a whole, regional differences were noted for consumption of instant noodles, packaged snacks or salted biscuits (Southern region, annual increase rate +1.05%, 95%CI 0.45; 1.66) and for consumption of filled cookies, sweets or treats (Northeast region, annual increase rate +2.94%, 95%CI 1.94; 3.95; and Southeast region, annual increase rate -1.66%, 95%CI -2.25; -1.06).

**Table 2:** Prevalence of consumption of unhealthy foods on the previous day and annual increase rate with 95% confidence intervals (95%CI) in adolescents, according to sex, age group and macro-region, in relation to Primary Health Care. Brazil, 2015-2019 (n=674,280)

	2015	2016	2017	2018	2019	Annual increase rate (95%CI)^a^	p-value	Trend^b^
	%	%	%	%	%			
**Consumption of hamburgers and sausages**								
Brazil	40.0	39.0	43.0	45.0	45.0	4.20 (2.18; 6.26)	0.007	↗
Sex								
Male	42.0	41.0	47.0	47.0	47.0	4.81 (1.97; 7.73)	0.012	↗
Female	40.0	38.0	42.0	43.0	43.0	3.72 (1.79; 5.67)	0.008	↗
**Age group (years)**								
10-14	40.0	39.0	44.0	45.0	45.0	4.62 (2.57; 6.70)	0.005	↗
15-19	40.0	39.0	43.0	44.0	44.0	3.72 (1.71; 5.76)	0.009	↗
Macro-region								
North	34.0	29.0	33.0	35.0	35.0	3.52 (-1.42; 8.69)	0.110	↔
Northeast	41.0	37.0	39.0	41.0	43.0	2.37 (-2.57; 7.55)	0.229	↔
Southeast	42.0	44.0	44.0	45.0	47.0	2.30 (1.71; 2.89)	0.001	↗
South	49.0	52.0	55.0	56.0	55.0	3.01 (-0.62; 6.78)	0.078	↔
Midwest	45.0	38.0	38.0	43.0	46.0	1.94 (-7.46; 12.28)	0.573	↔
**Consumption of sugar-sweetened drinks**								
Brazil	67.0	66.0	67.0	66.0	65.0	-0.47 (-1.16; 0.23)	0.120	↔
Sex								
Male	67.0	66.0	68.0	68.0	66.0	0.15 (-1.00; 1.31)	0.707	↔
Female	66.0	65.0	66.0	65.0	64.0	-0.85 (-1.36; -0.33)	0.014	↘
**Age group (years)**								
10-14	67.0	66.0	67.0	67.0	66.0	-0.02 (-0.78; 0.75)	0.948	↔
15-19	67.0	65.0	66.0	64.0	63.0	-1.05 (-1.81; -0.28)	0.023	↘
Macro-region								
North	66.0	63.0	60.0	60.0	58.0	-2.66 (-3.96; -1.35)	0.008	↘
Northeast	60.0	60.0	59.0	61.0	62.0	0.92 (-0.67; 2.52)	0.164	↔
Southeast	72.0	71.0	70.0	69.0	68.0	-1.46 (-1.64; -1.28)	0.000	↘
South	75.0	73.0	72.0	69.0	69.0	-2.12 (-2.44; -1.79)	0.001	↘
Midwest	74.0	67.0	66.0	68.0	70.0	-0.93 (-5.52; 3.87)	0.574	↔
**Consumption of instant noodles, packaged snacks or salted biscuits**								
Brazil	49.0	44.0	44.0	45.0	46.0	-0.78 (-5.17; 3.82)	0.620	↔
Sex								
Male	49.0	44.0	45.0	46.0	47.0	-0.31 (-4.66; 4.24)	0.839	↔
Female	49.0	44.0	43.0	45.0	46.0	-1.03 (-5.39; 3.54)	0.519	↔
**Age group (years)**								
10-14	51.0	46.0	46.0	47.0	49.0	-0.52 (-5.01; 4.19)	0.745	↔
15-19	47.0	43.0	42.0	43.0	44.0	-1.31 (-5.05; 2.59)	0.359	↔
Macro-region								
North	48.0	42.0	39.0	43.0	42.0	-2.45 (-9.18; 4.77)	0.349	↔
Northeast	51.0	47.0	47.0	49.0	49.0	-0.36 (-4.08; 3.51)	0.784	↔
Southeast	46.0	43.0	44.0	44.0	43.0	-0.94 (-2.47; 0.60)	0.147	↔
South	46.0	45.0	47.0	47.0	47.0	1.05 (0.45; 1.66)	0.011	↗
Midwest	52.0	42.0	40.0	41.0	46.0	-2.63 (-12.12; 7.88)	0.468	↔
**Consumption of filled cookies, sweets or treats**								
Brazil	56.0	54.0	56.0	55.0	55.0	0.15 (-0.48; 0.79)	0.499	↔
Sex								
Male	56.0	54.0	57.0	56.0	55.0	0.63 (-0.74; 2.03)	0.240	↔
Female	55.0	54.0	55.0	54.0	54.0	-0.14 (-0.61; 0.33)	0.408	↔
**Age group (years)**								
10-14	59.0	58.0	59.0	59.0	59.0	0.28 (-0.23; 0.79)	0.177	↔
15-19	52.0	51.0	53.0	51.0	51.0	-0.44 (-1.47; 0.59)	0.267	↔
Macro-region								
North	56.0	51.0	47.0	49.0	48.0	-3.39 (-7.13; 0.50)	0.069	↔
Northeast	50.0	50.0	52.0	54.0	54.0	2.94 (1.94; 3.95)	0.003	↗
Southeast	60.0	58.0	58.0	56.0	56.0	-1.66 (-2.25; -1.06)	0.007	↘
South	56.0	57.0	60.0	58.0	59.0	1.01 (-0.89; 2.94)	0.191	↔
Midwest	64.0	55.0	55.0	55.0	57.0	-2.39 (-7.14; 2.60)	0.220	↔
**Consumption of ultraprocessed food** ^c^								
Brazil	19.0	17.0	21.0	22.0	23.0	7.24 (2.95; 11.71)	0.012	↗
Sex								
Male	20.0	18.0	23.0	24.0	24.0	8.18 (3.43; 13.15)	0.011	↗
Female	18.0	17.0	19.0	21.0	22.0	6.55 (2.11; 11.18)	0.018	↗
**Age group** (years)								
10-14	20.0	19.0	22.0	24.0	24.0	7.69 (3.83; 11.69)	0.008	↗
15-19	18.0	16.0	19.0	20.0	21.0	6.40 (1.95; 11.05)	0.019	↗
Macro-region								
North	17.0	12.0	13.0	14.0	15.0	-0.02 (-12.72; 14.53)	0.997	↔
Northeast	18.0	15.0	17.0	20.0	22.0	7.43 (-2.39; 18.25)	0.098	↔
Southeast	19.0	21.0	23.0	24.0	23.0	5.00 (0.69; 9.50)	0.034	↗
South	21.0	23.0	27.0	28.0	29.0	9.08 (3.72; 14.72)	0.012	↗
Midwest	26.0	17.0	17.0	20.0	23.0	0.02 (-18.16; 22.25)	0.997	↔
Total	50.570	90.374	109.291	188.197	235.848			

^a^Annual increase rate calculated using the following formula [-1+(10^β^)]×100, where β is the Prais-Winsten regression coefficient; 95%CI calculated using the formulae [–1+10^βmin^]x100 e [–1+10^βmax^]x100; ^b^Trend: (↔) stationary trend, (↗) rising trend, when annual increase rate positive and p-value<0,05; and (↘) falling trend, when annual increase rate negative and p-value<0,05; ^c^Consumption of de ultraprocessed food: taking those individuals who answered “yes” to all four unhealthy eating markers (sausages, sugar-sweetened drinks, instant noodles, salted biscuits, sweets, treats and filled cookies).

Regarding the combination of ultra-processed food consumption, there was a consistently increasing temporal trend in the country (annual increase rate +7.24%, 95%CI 2.95; 11.71), rising from 19.0% in 2015 to 23.0% in 2019. There was a rising trend in both sexes and age groups. The Southeast region (annual increase rate +5.00%, 95%CI 0.69; 9.50) and the Southern region (annual increase rate +9.08%, 95%CI 3.72; 14.72) showed a rising trend, with 23.0% and 29.0% in 2019 (Table 2).

## Discussion

The findings of this study showed an increase in combined consumption of beans, fruit, vegetables and legumes in adolescents of both sexes, among those over 15 years old and those resident in the Southeast. This also occurred for ultra-processed foods, especially hamburgers and sausages. Most participants consumed sugar-sweetened beverages the day before. Despite this stable prevalence in the period in general in the country as a whole, significant reductions were observed among girls, those over 15 years old and residents of the North, South and Southeast regions.

The increase in prevalence of ultra-processed food consumption has been observed in national surveys conducted among adolescents (14-16). There was lower consumption of unprocessed and minimally processed foods among families with lower socioeconomic status (16). An inverse relationship between ultra-processed food consumption and daily fruit and vegetable intake was observed among Brazilian students (17). Ultra-processed foods accounted for more than half of the total daily caloric intake, and half of the young people reported consuming them more than three times a week (17). Daily consumption of the meals offered by the National School Meals Program was positively associated with the three markers of healthy eating composed of consumption of beans, fruit, and vegetables, when compared with non-adherence to school meals (OR 1.25; 95%CI 1.15; 1.36) (18).

The most prevalent consumption marker among Brazilian adolescents is beans, with emphasis on the Southeast, Midwest and Northeast regions (19). Prevalence of bean consumption decreased, between 2009 and 2019, from 62.0% to 54.0%, being higher among adolescents in state schools when compared to private schools (14-15).

Adolescents presented low consumption of fruit, with prevalence rates ​​of 31.0% in 2009 and 28.0% in 2019, with the highest rate for bananas, with 15.0% (14-15.19). Compared to adults and the elderly, adolescents consumed smaller amounts of fruit, vegetables and legumes. Lower likelihood of weekly consumption of fresh fruit was observed in the Center-South of Brazil compared to the state capital cities of the North and Northeast regions (14). Weekly consumption of vegetables was higher in the capitals of the Center-South of Brazil, with the exception of the city of Rio de Janeiro, and was lower in the Northeast (14). Only in the Midwest were vegetables among the five most consumed foods (19).

Sugar-sweetened drinks were among the most consumed foods among ultra-processed foods, especially among young people in the Southern region (19). Although there was a reduction in excessive consumption of sweetened drinks among young adults in the North and Northeast regions (19) and stability in soda consumption (15), among young people these foods continued to be high

Although the trend in sugar-sweetened drink consumption was stable on the SISVAN at the national level, a reduction in sugar-sweetened drink consumption was observed for some stratifications in this study: females, age group between 15-19 years old, and North, Southeast, and South regions. There was a reduction in weekly soft drink consumption equal to or greater than five days between 2009 and 2019, but access to cafeterias or alternative soft drink sales outlets was a concern, as 80.0% of 9^th^ grade elementary school students in Brazilian state capital cities reported this form of access, regardless of attending state or private schools (14).

The factors that influence food choices are multifactorial, such as price, availability, food environment, cultural issues, and behavioral and parental aspects, such as knowledge about food, culinary skills for food preparation, and time availability (5). Parents’ dietary habits, including consumption of sugary drinks, positively influence consumption of these beverages by their children (7,20-21). Parents’ attitudes toward consumption, children’s insistence behaviors, and perceived social acceptability of soft drinks are also considered influential factors for children’s consumption of sugary drinks. Effective interventions to reduce the consumption of these products should help parents deal with their children asking for them (22).

Healthy eating habits, such as a diet rich in vegetables, fruit, whole grains, fiber and healthy fats, were associated with better health conditions. Healthy diets were inversely related to plasma concentrations of markers of inflammatory potential, such as adiponectin (β=-0.177, p-value<0.050 and β=0.183), Interleukin-6 (p-value<0.050) and C-reactive protein (β=-0.09; β=0.04) (23,25) and reduction in the mean concentration of triglycerides (mean: 72.6, standard deviation 8.27, p-value 0.032) (25). Children and adolescents with unhealthy diets had higher fasting glucose (4.53 mmol/L vs. 4.46 mmol/L, p=0.0082), cholesterol LDL (2.156 mmol/L vs. 2.07 mmol/L, p=0.0023) and lower cholesterol HDL (p-value<0.001) concentrations compared to healthy dietary patterns (25). Dietary patterns rich in ultra-processed food were positively associated with systolic (β=0.469, p-value<0.001) and diastolic blood pressure (β=0.615, p-value<0.001), (25), higher prevalence of metabolic syndrome (OR 1.90; 95%CI 1.14), higher body mass index (0.94 kg/m^2^; 95%CI 0.42; 1.47), abdominal obesity (OR 1.62; 95%CI 1.39; 1.89) (26) and higher probability of developing childhood obesity (OR 2.2; 95%CI 1.4; 3.4) (24).

The World Health Organization reinforces the contribution of fiscal policies, such as taxation on sugary drinks and subsidies on fruits and vegetables, to changing food purchases and consumption (27-29). In Brazil, the front-of-pack nutritional labeling standard was implemented in 2022, and the reformulation of the basic food basket (*cesta básica*) with zero taxation rates for several products, including tax-exempt items that respect regional food culture, was approved in December 2024 (30).

The data produced by this study can be related to national surveys and studies that have assessed prevalence and trends in food consumption among Brazilian adolescents. The information generated by the SISVAN has not been fully explored for planning, management and evaluation of nutrition services in PHC in the Brazilian National Health System. Valuing adequate data recording legitimizes the importance of healthy eating as a cross-cutting theme in basic health actions (31). SISVAN is a powerful tool to assist PHC professionals in assessing the population’s food consumption. The protocols for using the Dietary Guidelines for the Brazilian Population can help health professionals apply its recommendations in individual consultations (32-34).

Data on population coverage of consumption marker records, as well as their percentage use, corroborate the potential for expansion in the production of food and nutrition surveillance data. Between 2015 and 2019, population coverage of food consumption marker records showed an annual increase rate of 45.63%, reaching 0.92% in 2019, with markers being used in more than half of Brazilian municipalities. Among adolescents, coverage ranged from 0.15% to 0.76%, with an annual increase rate of 49.0%. These data may be relevant for complementing longitudinal school surveys, with evidence for the local level (35). It is emphasized that food and nutrition surveillance actions should be carried out by any adequately qualified PHC health professional (36). It is essential to promote continuous reinforcement among PHC teams and emphasize the importance of their actions for diagnosis, patient guidance, and supporting public administration, in order to inform health promotion actions.

This study sought to analyze data collected by PHC teams monitoring the health of Brazilian adolescents, with relevant information for regional health service managers and public policy makers to inform health promotion actions. Some limitations are recognized, such as use of secondary data obtained from various sources, the low population coverage of the SISVAN food consumption markers registry, and the availability of few time points for temporal analysis, despite a certain statistical robustness for the short period evaluated.

Despite its limitations, this study identified relevant trends and regional differences in adolescent food consumption between 2015 and 2019. Brazilian adolescent food consumption during the period showed stability for beans and vegetables and legumes in all stratifications. There was a rising trend in combined consumption of beans, fruit, vegetables and legumes; in healthy consumption, but also a rising trend in combined consumption of ultra-processed foods, particularly hamburgers and sausages.

The high prevalence of ultra-processed food consumption and the low prevalence of consumption considered healthy point to significant challenges in promoting healthy eating habits at this stage of life and highlight the urgency of effective strategies to address the challenges identified. Implementation and strengthening of public policies can play a crucial role in promoting healthy food choices and, consequently, in improving the health of the population. The emphasis on data consolidated on the SISVAN highlights the continuous need to strengthen PHC teams in order to support effective health promotion actions at individual and collective levels.

## Data Availability

Public data, available at the Brazilian Health Ministry General Coordination of Food and Nutrition, provided upon request.

## References

[1] Ambrosini GL, Johns DJ, Northstone K, Emmett PM, Jebb SA (2015). Free sugars and total fat are important characteristics of a dietary pattern associated with adiposity across childhood and adolescence. J Nutr.

[2] Keats EC, Rappaport AI, Shah S, Oh C, Jain R, Bhutta ZA (2018). The dietary intake and practices of adolescent girls in low- and middle-income countries: a systematic review. Nutrients.

[3] Beal T, Morris SS, Tumilowicz A (2019). Global patterns of adolescent fruit, vegetable, carbonated soft drink, and fast-food consumption: a meta-analysis of global school-based student health surveys. Food Nutr Bull.

[4] Lane MM, Gamage E, Du S, Ashtree DN, McGuinness AJ, Gauci S (2024). Ultra-processed food exposure and adverse health outcomes: umbrella review of epidemiological meta-analyses. BMJ.

[5] Calcaterra V, Cena H, Magenes VC, Vincenti A, Comola G, Beretta A (2023). Sugar-sweetened beverages and metabolic risk in children and adolescents with obesity: a narrative review. Nutrients.

[6] Khan RK, Siraj MA, Kheya HR, Khalid S, Tabassum M, Zaman SB (2021). Consumption of sugar-sweetened beverages and their health impact on children. Discoveries.

[7] Mesas AE, González AD, Andrade SM, Martínez-Vizcaíno V, López-Gil JF, Jiménez-López E (2022). Increased consumption of ultra-processed food is associated with poor mental health in a nationally representative sample of adolescent students in Brazil. Nutrients.

[8] Popkin BM, Corvalan C, Grummer-Strawn LM (2020). Dynamics of the double burden of malnutrition and the changing nutrition reality. Lancet.

[9] World Health Organization (2017). Department of Nutrition for Health and Development.

[10] Brasil (2015). Orientações para avaliação de marcadores de consumo alimentar na atenção básica.

[11] Lourenço BH, Guedes BM, Santos TSS (2023). Marcadores do consumo alimentar do Sisvan: estrutura e invariância de mensuração no Brasil. Rev Saude Publica.

[12] Antunes JLF, Cardoso MRA (2015). Uso da análise de séries temporais em estudos epidemiológicos. Epidemiol Serv Saúde.

[13] Conselho Nacional de Saúde (2024). Resolução nº 738, de 1º de novembro de 2024.

[14] Instituto Brasileiro de Geografia e Estatística (2022). Pesquisa Nacional de Saúde do Escolar: análise de indicadores comparáveis dos escolares do 9º ano do ensino fundamental –municípios das capitais 2009/2019 [Internet].

[15] Instituto Brasileiro de Geografia e Estatística (2011). Pesquisa de Orçamentos Familiares 2008-2009: análise do consumo alimentar pessoal no Brasil [Internet].

[16] Silva MSL, Edwards B, Lima YMM, Ramalho AA (2022). Tendência temporal do consumo excessivo de refrigerantes e sucos artificiais nas capitais brasileiras e Distrito Federal (2007 a 2019). Desafios.

[17] Costa BGG, Duca GFD, Silva KS, Benedet J, Malheiros LEA, Quadros EN (2022). Socioeconomic inequalities in the consumption of minimally processed and ultra-processed foods in Brazilian adolescents. Cien Saude Colet.

[18] Froelich M, Souza BSN, Andrade ACS, Rodrigues PRM, Cunha DB, Muraro AP (2023). Adherence to school meals and co-occurrence of the healthy and unhealthy food markers among Brazilian adolescents. Cien Saude Colet.

[19] Souza AM, Barufaldi LA, Abreu GA, Giannini DT, Oliveira CL, Santos MM (2016). ERICA: ingestão de macro e micronutrientes em adolescentes brasileiros. Rev Saude Publica.

[20] Hebestreit A, Intemann T, Siani A, De Henauw S, Eiben G, Kourides YA (2017). Dietary patterns of European children and their parents in association with family food environment: results from the I.Family Study. Nutrients.

[21] Elfhag K, Tholin S, Rasmussen F (2008). Consumption of fruit, vegetables, sweets and soft drinks are associated with psychological dimensions of eating behaviour in parents and their 12-year-old children. Public Health Nutr.

[22] Pettigrew S, Jongenelis M, Chapman K, Miller C (2015). Factors influencing the frequency of children’s consumption of soft drinks. Appetite.

[23] Bujtor M, Turner AI, Torres SJ, Esteban-Gonzalo L, Pariante CM, Borsini A (2021). Associations of dietary intake on biological markers of inflammation in children and adolescents: a systematic review. Nutrients.

[24] Liberali R, Kupek E, Assis MAA (2020). Dietary Patterns and childhood obesity risk: a systematic review. Child Obes.

[25] Rocha NP, Milagres LC, Longo GZ, Ribeiro AQ, Novaes JF (2017). Association between dietary pattern and cardiometabolic risk in children and adolescents: a systematic review. J Pediatr.

[26] Elizabeth L, Machado P, Zinöcker M, Baker P, Lawrence M (2020). Ultra-processed foods and health outcomes: a narrative review. Nutrients.

[27] Bíró A (2015). Did the junk food tax make the Hungarians eat healthier?. Food Policy.

[28] Colchero MA, Rivera-Dommarco J, Popkin BM, Ng SW (2017). In Mexico, evidence of sustained consumer response two years after implementing a sugar-sweetened beverage tax. Health Aff.

[29] World Health Organization (2016). Fiscal policies for diet and the prevention of noncommunicable diseases [Internet].

[30] Agência Senado (2024). Reforma tributária: alimentos da cesta básica terão isenção; veja lista.

[31] Rolim MD, Lima SML, Barros DC, Andrade CLT (2015). Avaliação do Sisvan na gestão de ações de alimentação e nutrição em Minas Gerais, Brasil. Cien Saude Colet.

[32] Brasil (2013). Política Nacional de Alimentação e Nutrição.

[33] Brasil (2022). Guia para a organização da Vigilância Alimentar e Nutricional na Atenção Primária à Saúde [Internet].

[34] Brasil (2022). Fascículo 4: protocolo de uso do Guia Alimentar para a População Brasileira na Orientação Alimentar de crianças de 2 a 10 anos.

[35] Ricci JMS, Romito ALZ, Silva SA, Carioca AAF, Lourenço BH (2023). Marcadores do consumo alimentar do Sisvan: tendência temporal da cobertura e integração com o e-SUS APS, 2015-2019. Cien Saude Colet.

[36] Brasil (2004). Vigilância Alimentar e Nutricional (SISVAN): orientações básicas para a coleta, o processamento, a análise de dados e a informação em serviços de saúde [Internet].

