# Supplementary material for: Temporal trend of food consumption indicators of adolescents monitored by Primary Health Care services of the Brazilian National Health System, 2015-2019
Source: Epidemiol Serv Saude. 2025 Aug 4;34:e20240893. doi: 10.1590/S2237-96222025v34e20240893.en (PMC12334226; doi:10.1590/S2237-96222025v34e20240893.en)
Supplement: Translation

# Translation: Tendência temporal de indicadores do consumo alimentar de adolescentes acompanhados pela Atenção Primária à Saúde do Sistema Único de Saúde, 2015-2019

**DOI:** 10.1590/S2237-96222025v34e20240893.pt

**Authors:** Joana Brant de Carvalho, Andressa Freire Salviano, Bárbara Hatzlhoffer Lourenço, Luciana Yuki Tomita

**Affiliations:** 1 Universidade Federal de São Paulo, Escola Paulista de Medicina, Departamento de Medicina Preventiva, São Paulo, SP, Brasil; 2 Universidade de São Paulo, Faculdade de Saúde Pública, Programa de Pós-Graduação Nutrição em Saúde Pública, São Paulo, SP, Brasil; 3 Universidade de São Paulo, Faculdade de Saúde Pública, Departamento de Nutrição, São Paulo, SP, Brasil

**Keywords:** Consumo Alimentar, Adolescentes, Vigilância Alimentar e Nutricional, Sistemas de Informação em Saúde, Estudos de Séries Temporais

## Abstract

**Objetivo:**

Avaliar a tendência temporal de práticas alimentares de adolescentes acompanhados na Atenção Primária à Saúde no período 2015-2019.

**Métodos:**

Trata-se de estudo de séries temporais com microdados de marcadores do consumo alimentar de adolescentes (n=674.280 registros, média de idade: 14,9 [desvio-padrão: 3,0] anos, 65,0% meninas) no Sistema de Vigilância Alimentar e Nutricional. Calcularam-se prevalências anuais de cada marcador, além dos indicadores: consumo combinado de feijão, frutas, legumes e verduras; e alimentos ultraprocessados. Utilizou-se regressão de Prais-Winsten com cálculo da taxa de incremento anual, segundo sexo, faixa etária (10-14 anos, 15-19 anos) e macrorregiões. Tendências crescentes ou decrescentes foram denotadas por taxa de incremento anual positiva ou negativa com p-valor<0,05.

**Resultados:**

As prevalências do consumo de feijão, frutas, verduras e legumes em 2019 foram 83,0%, 69,0% e 65,0%. O indicador combinado de feijão, frutas, legumes e verduras ocorreu em 48,0% dos adolescentes em 2019, com tendência crescente no país (taxa de incremento anual: +4,0%, intervalo de confiança de 95% [IC95% 0,4; 6,6]), entre 15-19 anos de idade e na região Sudeste. Em 2019, o consumo de marcadores de alimentos ultraprocessados foi frequente entre adolescentes (hambúrgueres e embutidos: 45,0%, bebidas adoçadas: 65,0%, macarrão instantâneo/salgadinhos: 46,0%, doces: 55,0%). A prevalência do indicador de ultraprocessados em 2019 foi 23,0%, com tendência crescente nacionalmente (taxa de incremento anual: +7,0%, IC95% 2,9; 11,7) e nas macrorregiões Sul e Sudeste.

**Conclusão:**

Ainda que alguns indicadores saudáveis tenham aumentado, a prevalência de consumo de ultraprocessados entre adolescentes é elevada.

**Aspectos éticos**

A presente pesquisa respeitou os princípios éticos, obtendo os seguintes dados de aprovação: :

Comitê de ética em pesquisa: Universidade de São Paulo

Número do parecer: 4.172.787

Data de aprovação: 24/7/2020

Certificado de apresentação de apreciação ética: 35480520.2.0000.5421

Registro do consentimento livre e esclarecido: Dispensado.

## Introdução

O consumo de quantidades insuficientes de frutas, hortaliças e feijão e de quantidades excessivas de alimentos ultraprocessados, como refrigerantes, doces e guloseimas, salgadinhos e fast-food, tem sido observado nas últimas décadas entre adolescentes. Esse padrão aponta para o risco de substituição das dietas tradicionais pelas dietas obesogênicas, pobres em fibras e com alta densidade calórica, sódio, gordura e açúcar adicionado (1-3). Evidenciou-se a associação entre a ingestão de alimentos ultraprocessados e o maior risco de eventos adversos à saúde. O consumo desses alimentos foi relacionado a mais de 30 problemas de saúde, especialmente cardiometabólicos, incluindo obesidade, diabetes, hipertensão, dislipidemia, além de sono insatisfatório, câncer, outras doenças crônicas e mortalidade por todas as causas (4). Em crianças e adolescentes, foi observada correlação com menarca precoce, problemas de saúde mental e cáries dentárias (5-7). Isso afeta principalmente países de baixa e média renda, sendo um desafio de saúde pública global (8-9). 

No Brasil, o monitoramento do consumo alimentar da população é realizado continuamente pela Atenção Primária à Saúde (APS), por meio dos marcadores do consumo alimentar do Sistema de Vigilância Alimentar e Nutricional (Sisvan) (10). Esses marcadores são instrumentos para a triagem de práticas alimentares e ingestão de grupos de alimentos e avaliam o consumo alimentar do dia anterior para evitar vieses de memória. É uma ferramenta apropriada em nível individual para orientação da produção do cuidado em saúde por diferentes profissionais de saúde. Em nível populacional, a disponibilidade dessas informações permite o acompanhamento de aspectos qualitativos centrais da dieta ao longo do tempo, podendo apoiar a proposição de políticas para promoção da saúde (10). 

Diante dos riscos associados à má alimentação, este estudo teve como objetivo avaliar a tendência temporal de indicadores do consumo alimentar de adolescentes brasileiros assistidos na APS registrados no Sisvan. Houve interesse em explorar combinações de marcadores do consumo alimentar e investigar variações segundo sexo, recortes etários e regiões brasileiras.

## Métodos

### Delineamento do estudo e fontes de dados

Este estudo de séries temporais utilizou microdados a partir da agregação das informações individuais de marcadores do consumo alimentar do Sisvan. Foram utilizados 674.280 registros de adolescentes (10-19 anos de idade completos) entre 2015 e 2019. O período utilizado compreendeu o ano de inclusão do formulário de marcadores do consumo alimentar à luz das recomendações do *Guia Alimentar para a População Brasileira* no Sisvan, até o momento pré-pandêmico e época de solicitação das bases no início da vigência do projeto de pesquisa.

Os marcadores do consumo alimentar foram propostos com base nas recomendações do *Guia Alimentar para a População Brasileira* e incluíram nove questões sobre práticas alimentares e ingestão de sete grupos alimentares. Para este estudo, foram utilizados apenas os dados sobre consumo alimentar, com possíveis respostas “sim”, “não” ou “não sabe” para o consumo no dia anterior dos seguintes alimentos: feijão; frutas frescas excluindo os sucos de frutas; verduras e legumes, exceto tubérculos; hambúrgueres e embutidos; bebidas adoçadas; macarrão instantâneo, salgadinhos de pacote ou biscoitos salgados; biscoitos recheados, doces ou guloseimas (10). A estrutura interna do formulário de marcadores do consumo alimentar do Sisvan apresentou evidências de validade para avaliação de dois fatores da alimentação (saudável e não saudável), com estabilidade das características de mensuração entre macrorregiões brasileiras e grupos etários ao longo do tempo (11).

### Gerenciamento de dados e construção de indicadores

Os bancos de dados foram submetidos à etapa de limpeza, na qual foram identificados e excluídos os dados duplicados de um mesmo indivíduo em um mesmo município e no mesmo dia, dados do mesmo indivíduo em municípios diferentes e dados inconsistentes como idade negativa ou fase da vida não correspondente ao formulário respondido. Após a limpeza dos dados, foi considerado apenas o primeiro registro do ano de cada adolescente. No período 2015-2019, de 990.602 registros na faixa etária de interesse, excluíram-se 74.536 registros duplicados ou inconsistentes. Com a manutenção de apenas um registro por adolescente por ano, eliminaram-se 241.786 observações, o que resultou em 674.280 registros para as análises de séries temporais (Figura 1). 

**Figura 1:** Limpeza dos dados de marcadores do consumo alimentar de adolescentes com relação à Atenção Primária à Saúde. Brasil, 2015-2019

Calcularam-se as prevalências de adolescentes com respostas positivas aos marcadores no dia anterior, de 2015 a 2019. Além dos marcadores, foram criados indicadores do consumo alimentar através de agrupamentos: (i) consumo combinado de feijão, frutas, legumes e verduras; (ii) consumo de alimentos ultraprocessados (embutidos, bebidas adoçadas, macarrão instantâneo, biscoitos salgados, doces, guloseimas e biscoitos recheados); e (iii) consumo saudável, em que foram considerados aqueles indivíduos que consumiram todos os marcadores de alimentação saudável e nenhum marcador não saudável no dia anterior, à luz das orientações do *Guia Alimentar para a População Brasileira*: “fazer de alimentos in natura ou minimamente processados a base da alimentação” e “evitar alimentos ultraprocessados”.

### Análise de dados

A análise de dados foi realizada por regressão de Prais-Winsten, com cálculo da taxa de incremento anual das prevalências dos marcadores e indicadores do consumo alimentar entre adolescentes. Essa abordagem considerou a correlação serial das medidas, ou seja, a dependência de uma medida seriada com seus próprios valores em momentos anteriores, comumente observada em dados populacionais. A partir dos coeficientes estimados na regressão de Prais-Winsten, a taxa de incremento anual e o intervalo de confiança (IC95%) foram obtidos pela fórmula (12):


$$\text{Taxa de incremento anual}=[–1+10^{\beta }]*100$$

IC95% mínimo=[–1+10β
mín]∗
100

IC95% máximo=[–1+10β
máx]∗
100

β=coeficiente da regressão de Prais-Winsten

Os dados foram analisados em nível nacional, segundo sexo (masculino, feminino), faixa etária (10-14 anos completos; 15-19 anos completos) e macrorregião brasileira (Norte, Nordeste, Centro-Oeste, Sul e Sudeste). Para tal, utilizou-se o programa Stata versão 17.0. A interpretação da tendência temporal se deu pelos valores de taxa de incremento anual e o nível de significância estatística: crescente quando os valores de taxa de incremento anual foram positivos, e decrescente, quando negativos, acompanhados pelo nível de significância estatística de 5,0%. Foram utilizadas setas para representar os resultados tendência temporal: “↗” quando crescente, “↘” quando decrescente e “↔” quando estacionária. 

O uso e gerenciamento das bases de dados utilizadas neste estudo estão em consonância com os critérios normativos da Resolução do Conselho Nacional de Saúde nº 738, de 7 de novembro de 2024 (13).

## Resultados

Foram obtidos 674.280 registros de adolescentes entre 2015 e 2019 acompanhados na APS, com idade média de 14,9 (desvio-padrão: 3,0) anos, sendo 65,0% do sexo feminino. A prevalência do consumo de feijão no dia anterior foi 83,0% em 2019 para o Brasil, sem variação temporal significante nacionalmente e segundo sexo, faixa etária ou região (Tabela 1). O consumo de frutas apresentou estabilidade no país e para ambos os sexos e grupos etários, com prevalência nacional de 69,0% em 2019. Houve redução significante apenas para o Norte (2015: 70,0%, 2019: 65,0%; taxa de incremento anual -1,43%, IC95% -2,28; -0,57), ao passo que se registrou aumento no Sudeste (2015: 65,0%, 2019: 68,0%; taxa de incremento anual +2,30%, IC95% 0,79; 3,84). Para o consumo de verduras e legumes, notaram-se ocorrências estacionárias em todas as estratificações investigadas, com prevalência geral de 65,0% em 2019 para o Brasil.

**Tabela 1:** Prevalência de consumo de alimentos saudáveis no dia anterior e taxa de incremento anual com intervalo de confiança de 95% (IC95%) em adolescentes, segundo sexo, faixa etária e macrorregião, com relação à Atenção Primária à Saúde. Brasil, 2015-2019 (n=674.280)

	2015	2016	2017	2018	2019	Taxa de incremento anual (IC95%)^a^	p-valor	Tendência^b^
	%	%	%	%	%			
**Consumo de feijão**								
Brasil	83,0	81,0	84,0	83,0	83,0	0,26 (-0,60; 1,14)	0,407	↔
Sexo								
Masculino	85,0	82,0	87,0	85,0	85,0	0,37 (-0,67; 1,43)	0,340	↔
Feminino	83,0	80,0	83,0	81,0	81,0	0,16 (-0,67; 1,00)	0,578	↔
**Faixa etária** (anos)								
10-14	83,0	80,0	84,0	82,0	82,0	0,25 (-0,74; 1,25)	0,480	↔
15-19	84,0	81,0	84,0	83,0	83,0	0,28 (-0,54; 1,10)	0,359	↔
Macrorregião								
Norte	77,0	69,0	75,0	73,0	72,0	-0,07 (-2,44; 2,37)	0,936	↔
Nordeste	86,0	83,0	84,0	84,0	85,0	-0,12 (-1,18; 0,95)	0,738	↔
Sudeste	87,0	86,0	89,0	87,0	86,0	0,22 (-0,96; 1,41)	0,604	↔
Sul	78,0	78,0	79,0	75,0	74,0	-1,46 (-3,52; 0,65)	0,114	↔
Centro-Oeste	88,0	83,0	83,0	86,0	89,0	0,64 (-2,53; 3,91)	0,571	↔
**Consumo de frutas**								
Brasil	68,0	65,0	68,0	69,0	69,0	1,20 (-0,26; 2,68)	0,079	↔
Sexo								
Masculino	67,0	64,0	68,0	68,0	68,0	1,32 (-0,10; 2,76)	0,060	↔
Feminino	68,0	65,0	69,0	69,0	69,0	1,15 (-0,36; 2,67)	0,094	↔
**Faixa etária** (anos)								
10-14	68,0	65,0	68,0	68,0	68,0	1,07 (-0,31; 2,46)	0,090	↔
15-19	68,0	65,0	69,0	69,0	69,0	1,35 (-0,20; 2,93)	0,070	↔
Macrorregião								
Norte	70,0	65,0	67,0	65,0	65,0	-1,43 (-2,28; -0,57)	0,013	↘
Nordeste	67,0	63,0	66,0	68,0	69,0	1,66 (-1,18; 4,57)	0,161	↔
Sudeste	65,0	64,0	67,0	69,0	68,0	2,30 (0,79; 3,84)	0,017	↗
Sul	66,0	70,0	76,0	71,0	70,0	1,43 (-3,58; 6,71)	0,438	↔
Centro-Oeste	73,0	65,0	68,0	69,0	71,0	0,45 (-3,40; 4,46)	0,738	↔
**Consumo de verduras e legumes**								
Brasil	63,0	62,0	67,0	66,0	65,0	1,23 (-0,99; 3,50)	0,177	↔
Sexo								
Masculino	62,0	61,0	67,0	66,0	64,0	1,71 (-1,22; 4,72)	0,162	↔
Feminino	64,0	63,0	67,0	66,0	65,0	0,99 (-0,87; 2,88)	0,190	↔
**Faixa etária** (anos)								
10-14	61,0	60,0	65,0	64,0	62,0	1,28 (-1,33; 3,96)	0,219	↔
15-19	65,0	64,0	68,0	68,0	67,0	1,42 (-0,28; 3,14)	0,077	↔
Macrorregião								
Norte	62,0	56,0	58,0	58,0	58,0	-0,71 (-3,21; 1,86)	0,441	↔
Nordeste	58,0	55,0	57,0	58,0	60,0	1,55 (-0,46; 3,59)	0,092	↔
Sudeste	68,0	68,0	71,0	71,0	70,0	1,21 (-0,11; 2,55)	0,062	↔
Sul	68,0	70,0	74,0	69,0	68,0	-0,23 (-4,18; 3,87)	0,865	↔
Centro-Oeste	71,0	68,0	69,0	73,0	72,0	1,10 (-1,25; 3,50)	0,236	↔
**Consumo combinado de feijão, frutas, verduras e legumes** ^c^								
Brasil	45,0	42,0	48,0	48,0	48,0	3,48 (0,41; 6,64)	0,036	↗
Sexo								
Masculino	45,0	42,0	49,0	49,0	48,0	4,10 (0,52; 7,80)	0,035	↗
Feminino	45,0	41,0	47,0	48,0	47,0	3,11 (0,21; 6,11)	0,042	↗
**Faixa etária** (anos)								
10-14	44,0	40,0	47,0	47,0	46,0	3,23 (-0,10; 6,67)	0,054	↔
15-19	46,0	42,0	48,0	50,0	50,0	3,86 (0,78; 7,03)	0,028	↗
Macrorregião								
Norte	42,0	33,0	38,0	36,0	37,0	-0,89 (-5,51; 3,97)	0,595	↔
Nordeste	44,0	38,0	41,0	42,0	45,0	1,97 (-3,92; 8,21)	0,374	↔
Sudeste	46,0	47,0	52,0	55,0	53,0	4,98 (2,73; 7,27)	0,006	↗
Sul	43,0	46,0	53,0	49,0	49,0	3,26 (-3,32; 10,28)	0,219	↔
Centro-Oeste	53,0	46,0	47,0	52,0	55,0	2,31 (-5,20; 10,41)	0,411	↔
**Consumo saudável** ^d^								
Brasil	5,0	6,0	7,0	7,0	7,0	8,34 (3,19; 13,74)	0,014	↗
Sexo								
Masculino	4,0	5,0	6,0	6,0	6,0	9,28 (0,60; 18,70)	0,042	↗
Feminino	5,0	6,0	7,0	7,0	7,0	8,13 (4,66; 11,71)	0,005	↗
**Faixa etária** (anos)								
10-14	4,0	5,0	6,0	6,0	6,0	9,27 (0,89; 18,35)	0,039	↗
15-19	6,0	6,0	7,0	7,0	8,0	8,29 (6,73; 9,88)	<0,001	↗
Macrorregião								
Norte	5,0	5,0	7,0	6,0	7,0	9,45 (0,15; 19,62)	0,048	↗
Nordeste	6,0	6,0	7,0	7,0	7,0	6,67 (4,10; 9,30)	0,004	↗
Sudeste	4,0	5,0	6,0	7,0	7,0	13,61 (10,52 ;16,79)	0,001	↗
Sul	4,0	6,0	6,0	5,0	5,0	1,46 (-13,30; 18,74)	0,788	↔
Centro-Oeste	5,0	6,0	8,0	9,0	7,0	9,23 (-7,31; 28,70)	0,186	↔
Total	50.570	90.374	109.291	188.197	235.848			

^a^Taxa de incremento anual calculada de acordo com a fórmula [-1+(10^β^)]×100, em que β é o coeficiente da regressão de Prais-Winsten; IC95% calculado pelas fórmulas [–1+10^βmín^]x100 e [–1+10^βmáx^]x100; ^b^Tendência: (↔) tendência estacionária, (↗) tendência crescente, quando taxa de incremento anual positiva e p-valor<0,05; e (↘) tendência decrescente, quando taxa de incremento anual negativa e p-valor<0,05; ^c^Consumo combinado de feijão, frutas, verduras e legumes: foram considerados aqueles indivíduos que responderam “sim” para os três marcadores de alimentação saudável (feijão, frutas, verduras e legumes); ^d^Consumo saudável: foram considerados aqueles indivíduos que responderam “sim” para todos os marcadores de alimentação saudável e “não” para todos os marcadores de alimentação não saudável.

Considerando os indicadores produzidos, o consumo combinado de feijão, frutas, verduras e legumes no dia anterior apresentou prevalências baixas, variando de 45,0% a 48,0% no Brasil entre 2015 e 2019, contudo com tendência crescente (taxa de incremento anual +3,48%, IC95% 0,41; 6,64). A tendência crescente foi observada entre adolescentes de ambos os sexos e a partir de 15 anos. Entre as regiões, apenas o Sudeste apresentou tendência crescente de 4,98% (IC95% 2,73; 7,27) e alcançou 53,0% em 2019. Agregando a ausência de alimentos ultraprocessados, o indicador de consumo saudável apresentou prevalências diminutas, porém crescentes no país, e variou de 5,0% em 2015 para 7,0% em 2019 (taxa de incremento anual +8,34%, IC95% 3,19; 13,74). A tendência temporal positiva foi semelhante entre sexos e grupos etários, bem como para as regiões Norte, Nordeste e Sudeste (Tabela 1).

Para os grupos de alimentos ultraprocessados (Tabela 2), o marcador do consumo de hambúrgueres e embutidos apresentou tendência temporal crescente entre adolescentes no país, chegando a 45,0% em 2019 (taxa de incremento anual +4,20%, IC95% 2,18; 6,26). De forma semelhante, houve tendência crescente em ambos os sexos, grupos etários e no Sudeste. O consumo de bebidas adoçadas apresentou estabilidade em nível nacional, com prevalência de 65,0% em 2019. Adolescentes do sexo feminino (taxa de incremento anual -0,85%, IC95% -1,36; -0,33) e residentes nas regiões Norte (taxa de incremento anual -2,66%, IC95% -3,96; -1,35), Sudeste (taxa de incremento anual -1,46%, IC95% -1,64; -1,28) e Sul (taxa de incremento anual -2,12%, IC95% -2,44; -1,79) exibiram redução na prevalência deste marcador. Apesar da tendência de estabilidade no país, diferenças regionais foram notadas para o consumo de macarrão instantâneo, salgadinhos de pacote ou biscoitos salgados (região Sul, taxa de incremento anual +1,05%, IC95% 0,45; 1,66) e para o consumo de biscoitos recheados, doces ou guloseimas (região Nordeste, taxa de incremento anual +2,94%, IC95% 1,94; 3,95; e região Sudeste, taxa de incremento anual -1,66%, IC95% -2,25; -1,06). 

**Tabela 2:** Prevalência de consumo de alimentos não saudáveis no dia anterior e taxa de incremento anual com intervalo de confiança de 95% (IC95%) em adolescentes, segundo sexo, faixa etária e macrorregião, com relação à Atenção Primária à Saúde. Brasil, 2015-2019 (n=674.280)

	2015	2016	2017	2018	2019	Taxa de incremento anual (IC95%)^a^	p-valor	Tendência^b^
	%	%	%	%	%			
**Consumo de hambúrgueres e embutidos**								
Brasil	40,0	39,0	43,0	45,0	45,0	4,20 (2,18; 6,26)	0,007	↗
Sexo								
Masculino	42,0	41,0	47,0	47,0	47,0	4,81 (1,97; 7,73)	0,012	↗
Feminino	40,0	38,0	42,0	43,0	43,0	3,72 (1,79; 5,67)	0,008	↗
**Faixa etária** (anos)								
10-14	40,0	39,0	44,0	45,0	45,0	4,62 (2,57; 6,70)	0,005	↗
15-19	40,0	39,0	43,0	44,0	44,0	3,72 (1,71; 5,76)	0,009	↗
Macrorregião								
Norte	34,0	29,0	33,0	35,0	35,0	3,52 (-1,42; 8,69)	0,110	↔
Nordeste	41,0	37,0	39,0	41,0	43,0	2,37 (-2,57; 7,55)	0,229	↔
Sudeste	42,0	44,0	44,0	45,0	47,0	2,30 (1,71; 2,89)	0,001	↗
Sul	49,0	52,0	55,0	56,0	55,0	3,01 (-0,62; 6,78)	0,078	↔
Centro-Oeste	45,0	38,0	38,0	43,0	46,0	1,94 (-7,46; 12,28)	0,573	↔
**Consumo de bebidas adoçadas**								
Brasil	67,0	66,0	67,0	66,0	65,0	-0,47 (-1,16; 0,23)	0,120	↔
Sexo								
Masculino	67,0	66,0	68,0	68,0	66,0	0,15 (-1,00; 1,31)	0,707	↔
Feminino	66,0	65,0	66,0	65,0	64,0	-0,85 (-1,36; -0,33)	0,014	↘
**Faixa etária** (anos)								
10-14	67,0	66,0	67,0	67,0	66,0	-0,02 (-0,78; 0,75)	0,948	↔
15-19	67,0	65,0	66,0	64,0	63,0	-1,05 (-1,81; -0,28)	0,023	↘
Macrorregião								
Norte	66,0	63,0	60,0	60,0	58,0	-2,66 (-3,96; -1,35)	0,008	↘
Nordeste	60,0	60,0	59,0	61,0	62,0	0,92 (-0,67; 2,52)	0,164	↔
Sudeste	72,0	71,0	70,0	69,0	68,0	-1,46 (-1,64; -1,28)	0,000	↘
Sul	75,0	73,0	72,0	69,0	69,0	-2,12 (-2,44; -1,79)	0,001	↘
Centro-Oeste	74,0	67,0	66,0	68,0	70,0	-0,93 (-5,52; 3,87)	0,574	↔
**Consumo de macarrão instantâneo, salgadinhos de pacote ou biscoitos salgados**								
Brasil	49,0	44,0	44,0	45,0	46,0	-0,78 (-5,17; 3,82)	0,620	↔
Sexo								
Masculino	49,0	44,0	45,0	46,0	47,0	-0,31 (-4,66; 4,24)	0,839	↔
Feminino	49,0	44,0	43,0	45,0	46,0	-1,03 (-5,39; 3,54)	0,519	↔
**Faixa etária** (anos)								
10-14	51,0	46,0	46,0	47,0	49,0	-0,52 (-5,01; 4,19)	0,745	↔
15-19	47,0	43,0	42,0	43,0	44,0	-1,31 (-5,05; 2,59)	0,359	↔
Macrorregião								
Norte	48,0	42,0	39,0	43,0	42,0	-2,45 (-9,18; 4,77)	0,349	↔
Nordeste	51,0	47,0	47,0	49,0	49,0	-0,36 (-4,08; 3,51)	0,784	↔
Sudeste	46,0	43,0	44,0	44,0	43,0	-0,94 (-2,47; 0,60)	0,147	↔
Sul	46,0	45,0	47,0	47,0	47,0	1,05 (0,45; 1,66)	0,011	↗
Centro-Oeste	52,0	42,0	40,0	41,0	46,0	-2,63 (-12,12; 7,88)	0,468	↔
**Consumo de biscoitos recheados, doces ou guloseimas**								
Brasil	56,0	54,0	56,0	55,0	55,0	0,15 (-0,48; 0,79)	0,499	↔
Sexo								
Masculino	56,0	54,0	57,0	56,0	55,0	0,63 (-0,74; 2,03)	0,240	↔
Feminino	55,0	54,0	55,0	54,0	54,0	-0,14 (-0,61; 0,33)	0,408	↔
**Faixa etária** (anos)								
10-14	59,0	58,0	59,0	59,0	59,0	0,28 (-0,23; 0,79)	0,177	↔
15-19	52,0	51,0	53,0	51,0	51,0	-0,44 (-1,47; 0,59)	0,267	↔
Macrorregião								
Norte	56,0	51,0	47,0	49,0	48,0	-3,39 (-7,13; 0,50)	0,069	↔
Nordeste	50,0	50,0	52,0	54,0	54,0	2,94 (1,94; 3,95)	0,003	↗
Sudeste	60,0	58,0	58,0	56,0	56,0	-1,66 (-2,25; -1,06)	0,007	↘
Sul	56,0	57,0	60,0	58,0	59,0	1,01 (-0,89; 2,94)	0,191	↔
Centro-Oeste	64,0	55,0	55,0	55,0	57,0	-2,39 (-7,14; 2,60)	0,220	↔
**Consumo de ultraprocessados** ^c^								
Brasil	19,0	17,0	21,0	22,0	23,0	7,24 (2,95; 11,71)	0,012	↗
Sexo								
Masculino	20,0	18,0	23,0	24,0	24,0	8,18 (3,43; 13,15)	0,011	↗
Feminino	18,0	17,0	19,0	21,0	22,0	6,55 (2,11; 11,18)	0,018	↗
**Faixa etária** (anos)								
10-14	20,0	19,0	22,0	24,0	24,0	7,69 (3,83; 11,69)	0,008	↗
15-19	18,0	16,0	19,0	20,0	21,0	6,40 (1,95; 11,05)	0,019	↗
Macrorregião								
Norte	17,0	12,0	13,0	14,0	15,0	-0,02 (-12,72; 14,53)	0,997	↔
Nordeste	18,0	15,0	17,0	20,0	22,0	7,43 (-2,39; 18,25)	0,098	↔
Sudeste	19,0	21,0	23,0	24,0	23,0	5,00 (0,69; 9,50)	0,034	↗
Sul	21,0	23,0	27,0	28,0	29,0	9,08 (3,72; 14,72)	0,012	↗
Centro-Oeste	26,0	17,0	17,0	20,0	23,0	0,02 (-18,16; 22,25)	0,997	↔
Total	50.570	90.374	109.291	188.197	235.848			

^a^Taxa de incremento anual calculada de acordo com a fórmula [-1+(10^β^)]×100, em que β é o coeficiente da regressão de Prais-Winsten; IC95% calculado pelas fórmulas [–1+10^βmín^]x100 e [–1+10^βmáx^]x100; ^b^Tendência: (↔) tendência estacionária, (↗) tendência crescente, quando taxa de incremento anual positiva e p-valor<0,05; e (↘) tendência decrescente, quando taxa de incremento anual negativa e p-valor<0,05; ^c^Consumo de ultraprocessados: considerados aqueles indivíduos que responderam “sim” para todos os quatro marcadores de alimentação não saudável (embutidos, bebidas adoçadas, macarrão instantâneo, biscoitos salgados, doces, guloseimas e biscoitos recheados).

Com relação à combinação do consumo de alimentos ultraprocessados, houve tendência temporal consistentemente crescente no país (taxa de incremento anual +7,24%, IC95% 2,95; 11,71), passando de 19,0% em 2015 para 23,0% em 2019. Houve tendência crescente em ambos os sexos e grupos etários. O Sudeste (taxa de incremento anual +5,00%, IC95% 0,69; 9,50) e o Sul (taxa de incremento anual +9,08%, IC95% 3,72; 14,72) apresentaram tendência crescente, com ocorrências de 23,0% e 29,0%, em 2019 (Tabela 2).

## Discussão

Os achados deste estudo mostraram aumento no consumo combinado de feijão, frutas, verduras e legumes em adolescentes de ambos os sexos, entre maiores de 15 anos e residentes do Sudeste. Isso também ocorreu para os alimentos ultraprocessados, especialmente hambúrgueres e embutidos. A maioria dos participantes consumiu bebidas adoçadas no dia anterior. Apesar desta prevalência estável no período de forma geral no país, reduções significativas foram observadas entre as meninas, nos maiores de 15 anos e nos residentes das regiões Norte, Sul e Sudeste.

O aumento na prevalência de consumo de alimentos ultraprocessados tem sido observado nos inquéritos nacionais conduzidos entre os adolescentes (14-16). Houve menor consumo de alimentos in natura e minimamente processados entre as famílias de menor nível socioeconômico (16). A relação inversa entre o consumo de alimentos ultraprocessados e a ingestão diária de frutas e vegetais foi observada entre os estudantes brasileiros (17). Alimentos ultraprocessados foram responsáveis por mais da metade da ingestão total calórica diária, e metade dos jovens reportou consumi-los mais de três vezes na semana (17). Realizar diariamente a refeição oferecida pelo Programa Nacional de Alimentação Escolar esteve positivamente associado aos três marcadores de alimentação saudável composto pelo consumo de feijão, frutas e hortaliças, quando comparado com a não adesão à alimentação escolar (OR 1,25; IC95% 1,15; 1,36) (18).

O marcador de consumo com maior prevalência entre adolescentes brasileiros é o feijão, com destaque para as regiões Sudeste, Centro-Oeste e Nordeste (19). A prevalência de consumo de feijão reduziu, entre 2009 e 2019, de 62,0% para 54,0%, sendo maior entre os adolescentes de escolas públicas quando comparadas a escolas privadas (14-15).

Adolescentes apresentaram baixo consumo de frutas, com valores de prevalências de 31,0% em 2009 e 28,0% em 2019, sendo o maior valor para a variedade banana, com 15,0% (14-15,19). Em comparação com os adultos e idosos, os adolescentes consumiram menores quantidades de frutas, verduras e legumes. Menores chances de consumo semanal de frutas frescas foram observadas no Centro-Sul em comparação às capitais do Norte e do Nordeste (14). O consumo semanal de legumes foi maior nas capitais do Centro-Sul brasileiro, com exceção do município do Rio de Janeiro, e foi menor no Nordeste (14). Somente no Centro-Oeste, as hortaliças ficaram entre os cinco alimentos mais consumidos (19). 

As bebidas adoçadas ficaram entre os alimentos mais consumidos entre os ultraprocessados, principalmente entre os jovens da região Sul (19). Enquanto observou-se redução no consumo excessivo de bebida adoçada entre os adultos jovens das regiões Norte e Nordeste (19) e estabilidade no consumo de refrigerante (15), entre os jovens esses alimentos permaneceram elevados. 

Embora tenha sido observada a estabilidade da tendência do consumo de bebidas adoçadas no Sisvan em nível nacional, observou-se a redução no consumo de bebidas adoçadas para algumas estratificações neste estudo: sexo feminino, faixa etária entre 15-19 anos completos e regiões Norte, Sudeste e Sul. Houve redução do consumo semanal de refrigerantes igual ou superior a cinco dias, entre 2009 e 2019, mas o acesso à cantina ou pontos de venda alternativos com disponibilidade de refrigerante foi preocupante, pois 80,0% dos alunos do 9º ano do ensino fundamental nas capitais brasileiras reportaram esse acesso, independentemente da rede de ensino (14). 

Os fatores que influenciam nas escolhas alimentares são multifatoriais como preço, disponibilidade, ambiente alimentar, questões culturais e os aspectos comportamentais e parentais, como o conhecimento em relação à alimentação, as habilidades culinárias para o preparo de alimentos e a disponibilidade de tempo (5). Os hábitos alimentares dos pais, incluindo o consumo de bebidas açucaradas, influenciam positivamente o consumo dessas bebidas pelos seus filhos (7,20-21). A atitude dos pais em relação ao consumo, os comportamentos de insistência das crianças e a aceitabilidade social percebida dos refrigerantes também são considerados fatores influentes para o consumo de bebidas açucaradas pelos filhos. Intervenções eficazes para a redução do consumo desses produtos devem auxiliar os pais a lidarem com os pedidos de seus filhos (22).

Hábitos alimentares saudáveis, como alimentação rica em hortaliças, frutas, cereais integrais, fibras e gorduras saudáveis, estavam associados a melhores condições de saúde. Dietas saudáveis foram inversamente relacionadas a concentrações plasmáticas de marcadores de potencial inflamatório, como adiponectina (β=-0,177, p-valor<0,050 e β=0,183), IL-6 (p-valor<0,050) e PCR (β=-0,09; β=0,04) (23,25) e redução na concentração média de triglicerídeos (média: 72,6, desvio-padrão 8,27, p-valor 0,032) (25). Crianças e adolescentes com dietas pouco saudáveis apresentaram concentrações mais altas de glicemia de jejum (4,53 mmol/L versus 4,46 mmol/L, p-valor 0,0082), LDL-colesterol (2,156 mmol/L vs. 2,07 mmol/L, p-valor 0,0023) e mais baixas de HDL-colesterol (p-valor<0,001) em comparação com padrões alimentares saudáveis (25). Padrões dietéticos ricos em ultraprocessados tiveram associação positiva com a pressão arterial sistólica (β=0,469, p-valor<0,001) e diastólica (β=0,615, p-valor<0,001), (25), maior prevalência de síndrome metabólica (OR 1,90; IC95% 1,14), maior IMC (0,94 kg/m^2^; IC95% 0,42; 1,47), obesidade abdominal (OR 1,62; IC95% 1,39; 1,89) (26) e maior probabilidade de desenvolvimento de obesidade infantil (OR 2,2; IC95% 1,4; 3,4) (24).

A Organização Mundial da Saúde reforça a contribuição das políticas fiscais, como a tributação em bebidas açucaradas e subsídios às frutas e hortaliças, para a mudança na compra e no consumo dos alimentos (27-29). No Brasil, a norma de rotulagem nutricional frontal foi implementada em 2022, e a reformulação da cesta básica com alíquota zero para diversos produtos, incluindo itens que respeitam a cultura alimentar regional isentos de impostos, foi aprovada em dezembro de 2024 (30).

Os dados deste estudo podem ser relacionados aos inquéritos e estudos nacionais que avaliaram as prevalências e tendências de consumo alimentar de adolescentes brasileiros. As informações geradas no Sisvan não têm sido exploradas em todo o seu potencial para o planejamento, a gestão e a avaliação dos serviços de nutrição na APS no Sistema Único de Saúde. Valorizar o registro adequado dos dados é legitimar a importância da alimentação saudável como tema transversal das ações básicas de saúde (31). O Sisvan é uma potente ferramenta para assistir os profissionais da APS na avaliação do consumo alimentar da população. Os protocolos de uso do Guia Alimentar para a População Brasileira podem auxiliar os profissionais de saúde a aplicarem as diretrizes do guia nas consultas individuais (32-34). 

Dados da cobertura populacional dos registros de marcadores do consumo, bem como seu percentual de utilização, corroboram o potencial de expansão na produção de dados de vigilância alimentar e nutricional. A cobertura populacional do registro de marcadores do consumo alimentar apresentou, entre 2015 e 2019, taxa de incremento anual de 45,63%, alcançando 0,92% em 2019, com utilização dos marcadores em mais da metade dos municípios brasileiros. Entre os adolescentes, a cobertura variou de 0,15% a 0,76%, com taxa de incremento anual de 49,0%. Esses dados podem ser relevantes para complementar longitudinalmente inquéritos escolares, com evidências para o nível local (35). Reforça-se que a ação da vigilância alimentar e nutricional deve ser realizada por qualquer profissional de saúde da APS, com adequada qualificação (36). É essencial promover reforço contínuo entre as equipes da APS e enfatizar a importância das suas ações para diagnóstico, orientação ao paciente e apoio à gestão pública, a fim de subsidiar ações de promoção da saúde. 

Este estudo se esforçou para analisar os dados coletados pelas equipes da APS no acompanhamento de saúde de adolescentes brasileiros, com informações relevantes aos gestores regionais e formuladores de políticas públicas para subsidiar ações de promoção da saúde. Reconhecem-se algumas limitações, como a utilização de dados secundários, obtidos de diversas fontes, a baixa cobertura populacional do registro dos marcadores de consumo alimentar do Sisvan e a disponibilidade de poucos pontos no tempo para análise temporal, apesar de certa robustez estatística para o curto período avaliado. 

Apesar das limitações, este estudo identificou tendências relevantes e diferenças regionais no consumo alimentar dos adolescentes entre 2015 e 2019. O consumo alimentar de adolescentes brasileiros no período demonstrou estabilidade para feijão e para verduras e legumes em todas as estratificações. Houve tendência crescente no consumo combinado de feijão, frutas, verduras e legumes; no consumo saudável, mas também no consumo combinado de ultraprocessados e particularmente de hambúrgueres e embutidos. 

A alta prevalência de consumo de alimentos ultraprocessados e a baixa prevalência do consumo considerado saudável aponta para desafios significativos na promoção de hábitos alimentares saudáveis nessa fase da vida e ressalta a urgência de estratégias efetivas para enfrentar os desafios identificados. A implementação e o fortalecimento de políticas públicas podem desempenhar papel crucial na promoção de escolhas alimentares saudáveis e, consequentemente, na melhoria da saúde da população. A ênfase em dados consolidados no Sisvan destaca a necessidade contínua de reforço nas equipes de APS para subsidiar ações eficazes de promoção da saúde em níveis individuais e coletivos.

## Data Availability

Dados públicos, disponíveis na Coordenação Geral de Alimentação e Nutrição do Ministério da Saúde, cedidos mediante solicitação.
